# Supplementary material for: EASY-NET Program: Effectiveness of an Audit and Feedback Intervention in the Emergency Care for Acute Conditions in the Lazio Region
Source: Healthcare (Basel). 2024 Mar 27;12(7):733. doi: 10.3390/healthcare12070733 (PMC11012083; doi:10.3390/healthcare12070733)
Supplement: Supplementary file 1 [file healthcare-12-00733-s001.zip › healthcare-2906729-supplementary.pdf]

Sommario

Table S1. Detailed cardiovascular area (AMI/STEMI) indicators sheets. 3

Table S2. Detailed cerebrovascular area indicators sheet. 3

Table S3. ICD-9-CM codes. 5

Table S4. List of participating facilities. 7

Figure S1. Flow chart of included Lazio facilities with AMI patients. 7

Figure S2. Flow chart of included Lazio facilities with STEMI patients. 8

Figure S3. Flow chart of included Lazio facilities with ischemic stroke patients. 8

Table S5. Distribution of patients belonging to the IMA cohorts by year of analysis and facility. 9

Table S6. Distribution of patients belonging to the STEMI cohorts by year of analysis 10

and facility 10

Table S7. Distribution of patients belonging to stroke cohorts by year of analysis and 11

facility (a and b). 11

Table S8. Characteristics of patients included in the AMI cohort in 2021 and 2022 from participating facilities according to A&F intervention exposure status. 12

Table S9. Characteristics of patients included in the AMI cohort in 2021 (PRE) from participating facilities by 30-days mortality after first hospital admission in patients with AMI (Yes/No) 12

Table S10. Characteristics of patients included in the AMI cohort in 2022 (POST) from participating facilities by 30-days mortality after first hospital admission in patients with AMI (Yes/No) 13

Table S11. Characteristics of patients included in the STEMI cohort in 2021 and 2022 from participating facilities according to A&F intervention exposure status. 15

Table S12. Characteristics of patients included in the STEMI cohort in 2021 (PRE) from participating facilities according to performing of PTCA within 90 minutes of admission to the hospital ER (Yes/No). 16

Table S13. Characteristics of patients included in the STEMI cohort in 2022 (POST) from participating facilities according to performing of PTCA within 90 minutes of admission to the hospital ER (Yes/No). 17

Table S14. Characteristics of patients included in the ischemic stroke cohort (in hospital mortality) in 2021 and 2022 from participating facilities according to A&F intervention exposure status. 18

Table S15. Characteristics of patients included in the ischemic stroke cohort in 2021 (PRE) from participating facilities by in hospital 30-days mortality since first hospital admission in patients with ischemic stroke (Yes/No). 19

Table S16. Characteristics of patients included in the ischemic stroke cohort in 2022 (POST) from participating facilities by in hospital 30-days mortality since first hospital admission in patients with ischemic stroke (Yes/No). 20

Table S17. Characteristics of patients included in the ischemic stroke cohort (hospital readmissions) in 2021 and 2022 from participating facilities according to A&F intervention exposure status. 21

Table S18. Characteristics of patients included in the stroke cohort in 2021 (PRE) from participating facilities according to in hospital readmissions within 30-days of discharge for ischemic stroke (Yes/No). 22

Table S19. Characteristics of patients included in the stroke cohort in 2022 (POST) from participating facilities according to in hospital readmissions within 30-days of discharge for ischemic stroke (Yes/No)..23

Table S1. Detailed cardiovascular area (AMI/STEMI) indicators sheets.

INDICATOR	**30-days mortality after first hospital admission of patients with AMI**
DIMENSION	Outcome
RATIONAL	Timely and effective treatments are essential for the survival of the acute myocardial infarction (AMI) patient, particularly in the case of a STEMI. Mortality at 30-days after IMA is considered a valid and reproducible indicator of the appropriateness and effectiveness of the diagnostic-therapeutic process that begins with hospitalization. With the widespread use of fibrinolytic drugs, aspirin, and coronary revascularization interventions, mortality has gone from 18% to 6-7% in the past 30 years. Reperfusion therapy, if implemented correctly and with the necessary timeliness, results in both decreased mortality and improved short- and long-term prognosis<br>However, the choice of treatment pathway should be made even before arrival at the hospital taking into account the characteristics of the available organization and the patient's clinical picture, based on which to perform risk stratification.<br>https://www.dep.lazio.it/prevale2022/documenti/razionali/definizione_1.pdf
YEAR/S	2021,2022
STANDARD	Nd; analysis by volume classes
PROTOCOL	https://www.dep.lazio.it/prevale2022/documenti/protocolli/pro_1.pdf

INDICATOR	**Proportion of PTCA performed in STEMI patients within 90 min of admission to the hospital ER**
DIMENSION	Timeliness
RATIONAL	In patients with STEMI, PTCA is considered the treatment of choice when it can be performed by expert personnel, in an appropriate Cath laboratory, within 90 minutes of the first contact with the Health Service. In fact, the literature has demonstrated the inverse relationship between the timeliness of execution of the procedure in patients with AMI (door-to-balloon time) and short-term mortality.<br>https://www.dep.lazio.it/prevale2022/documenti/razionali/definizione_610.pdf
YEAR/S	2021,2022
STANDARD	≥60% (DM 70/2015; DCA 412/2014)
PROTOCOL	https://www.dep.lazio.it/prevale2022/documenti/protocolli/pro_610.pdf

Table S2. Detailed cerebrovascular area indicators sheet.

INDICATOR	**30-days in hospital mortality after first hospital admission in patients with ischemic stroke**<br><br>**Proportion of in hospital readmissions within 30-days of discharge for ischemic stroke**
DIMENSION	Outcome
RATIONAL	Individuals with stroke are at increased risk of adverse outcomes following the acute event including death but also recurrence, disability, complications, and general clinical deterioration.

	Mortality at 30-days after hospitalization for stroke is internationally considered a valid and reproducible indicator of the appropriateness and effectiveness of the diagnostic-therapeutic process.<br>Given the likelihood of recurrence, "hospital readmissions at 30-days after treatment for ischemic stroke" also represents a short-term, measurable health outcome. Recurrences may be associated with disease progression but may also depend on the quality of both intra- and extrahospital care processes. The rate of rehospitalizations 30-days after a hospital discharge can, therefore, be a valid indicator of the quality of care provided. There is evidence in the literature that improvements in discharge processes can, for example, reduce the rate of hospital readmissions. These also expose the patient to additional risk of hospital infections and complications in general as well as incurring additional costs for hospitals. Monitoring this outcome offers the possibility of identifying opportunities for improvement in the care process, in this case intrahospital, and achieving better patient outcomes.<br>https://www.dep.lazio.it/prevale2022/documenti/razionali/definizione_18.pdf
YEAR/S	2021,2022
STANDARD	nd;
PROTOCOL	https://www.dep.lazio.it/prevale2022/documenti/protocolli/pro_19.pdf<br>https://www.dep.lazio.it/prevale2022/documenti/protocolli/pro_18.pdf

Table S3. ICD-9-CM codes.

Indicators	Calculation	Data sources	ICD9-CM primary or secondary diagnoses codes		ICD9-CM procedures codes
			Inclusion	Exclusion	Inclusion
STEMI: Proportion of PTCA performed in STEMI patients within 90 min of admission to the hospital ER	Number of hospitalized patients diagnosed with STEMI who received PTCA within 90 minutes from access to ER / Number of hospitalization of patients diagnosed with STEMI	HDR, HEIS	410.XX<br><br>411, 413, 414, 423.0, 426, 427, 428, 429.5, 429.6, 429.71, 429.79, 429.81, 518.4, 518.81, 780.01, 780.2, 785.51, 799.1, 997.02, 998.2	410.7X, 410.9.X 427.5	00.66, 36.01, 36.02, 36.05, 36.06 36.07
AMI: 30-days mortality after first hospital admission of patients with AMI	Number of hospitalized patients diagnosed with STEMI who died during the 30-days after hospital admission/ Number of hospitalized patients diagnosed with AMI	HDR, HEIS, Tax Registry			
Ischemic Stroke: intrahospital 30-days mortality after first hospital admission in patients with ischemic stroke	Number of admissions with a principal diagnosis of ischemic stroke in which the patient is found to have died within thirty days from the date of first admission/Number of hospitalizations with principal diagnosis of ischemic stroke.	HDR, HEIS, Tax Registry	433.x1, 434.x1, 436	430,431,432.X 290-319 140.0-208.9<br><br>Admissions preceded by another hospitalization with a stroke diagnosis within 1 year from the date of first admission (430,431,	72-75

				432.X, 433.x1, 434.x1, 436)	
Ischemic Stroke: in hospital readmissions within 30-days of discharge for ischemic stroke	Number of hospitalizations followed within 30-days from the date of discharge by at least one hospitalization ordinary acute care hospital admission/ Number hospitalizations with principal diagnosis of ischemic stroke.	HDR, HEIS,	433.x1, 434.x1, 436	430,431,432.X 290-319 140.0-208.9<br><br>Admissions preceded by another hospitalization with a stroke diagnosis within 1 year from the date of first admission (430,431, 432.X, 433.x1, 434.x1, 436)	72-75

STEMI: ST Elevation Myocardial Infarction
PTCA: Percutaneous Transluminal Coronary Angioplasty
ER: Emergency Room
HEIS: Healthcare Emergency Information System
HDR: Italian Hospital Discharge Registry

Table S4. List of participating facilities.

	**PATHWAYS**	
	**AMI**	**Ischemic stroke**
**Participating facilities**	Osp. S. Spirito (ASL Roma 1)	Osp. S. Spirito (ASL Roma 1)
	Osp. S. Eugenio-CTO (ASL Roma 2)	Osp. S. Eugenio-CTO (ASL Roma 2)
	Osp. G. Grassi (ASL Roma 3)	Osp. G. Grassi (ASL Roma 3)
	Osp. S. Paolo (ASL Roma 4)	Osp. S. Paolo (ASL Roma 4)
	Osp. L. Parodi Delfino (ASL Roma 5)	Osp. L. Parodi Delfino (ASL Roma 5)
	Nuovo Ospedale dei Castelli (ASL Roma 6)	
	Osp. S. Maria Goretti (ASL Latina)	Osp. S. Maria Goretti (ASL Latina)
	Osp. F. Spaziani (ASL Frosinone)	
	Osp. S. Camillo De Lellis (ASL Rieti)	Osp. S. Camillo De Lellis (ASL Rieti)
	Osp. Di Belcolle (ASL Viterbo)	Osp. Di Belcolle (ASL Viterbo)
	A.O.U.U. S. Andrea	A.O.U.U. S. Andrea
	Pol. U. A. Gemelli	Pol. U. A. Gemelli
	Osp. S. Carlo di Nancy	Osp. S. Carlo di Nancy
	A.O. S. Camillo Forlanini	
	Osp. S. Pertini	
	Osp. M.G. Vannini	
		A.O.U.U. Umberto I
		A.O.U.U. Tor Vergata

Figure S1. Flow chart of included Lazio facilities with AMI patients.

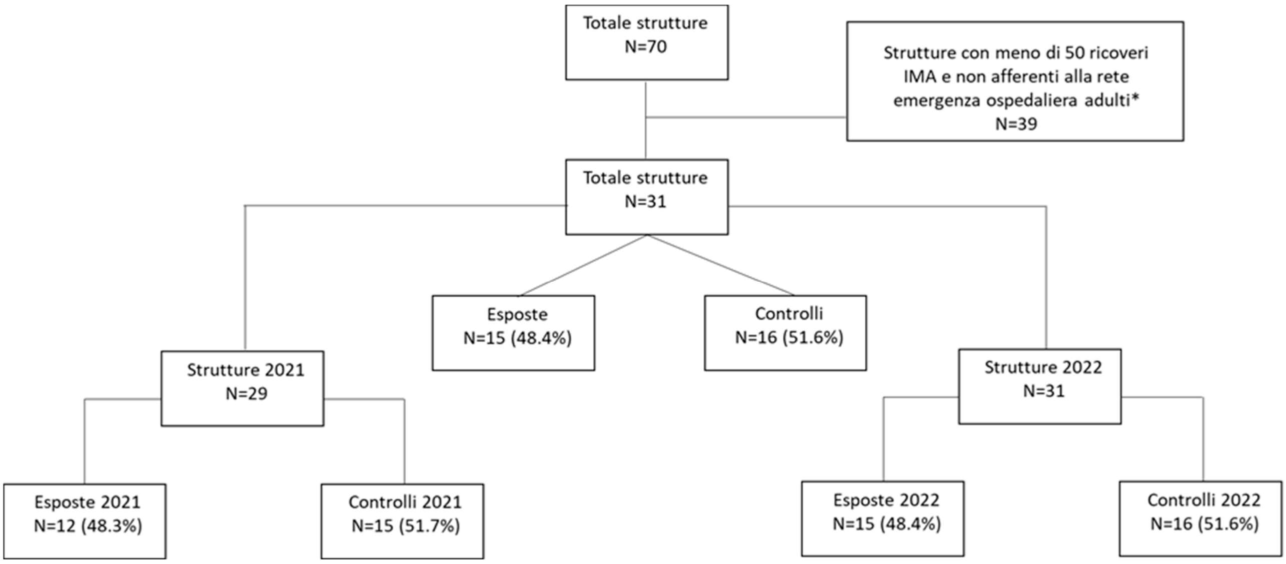

* https://www.regione.lazio.it/enti/salute/sistemi-emergenza/rete-emergenza-ospedaliera

Figure S2. Flow chart of included Lazio facilities with STEMI patients.

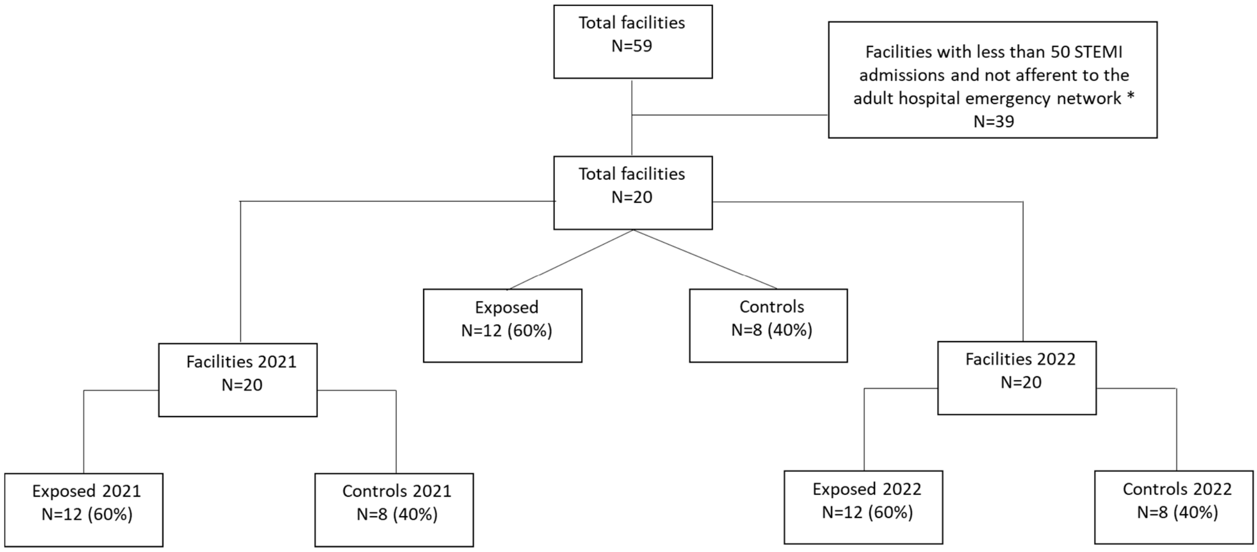

* https://www.regione.lazio.it/enti/salute/sistemi-emergenza/rete-emergenza-ospedaliera

Figure S3. Flow chart of included Lazio facilities with ischemic stroke patients.

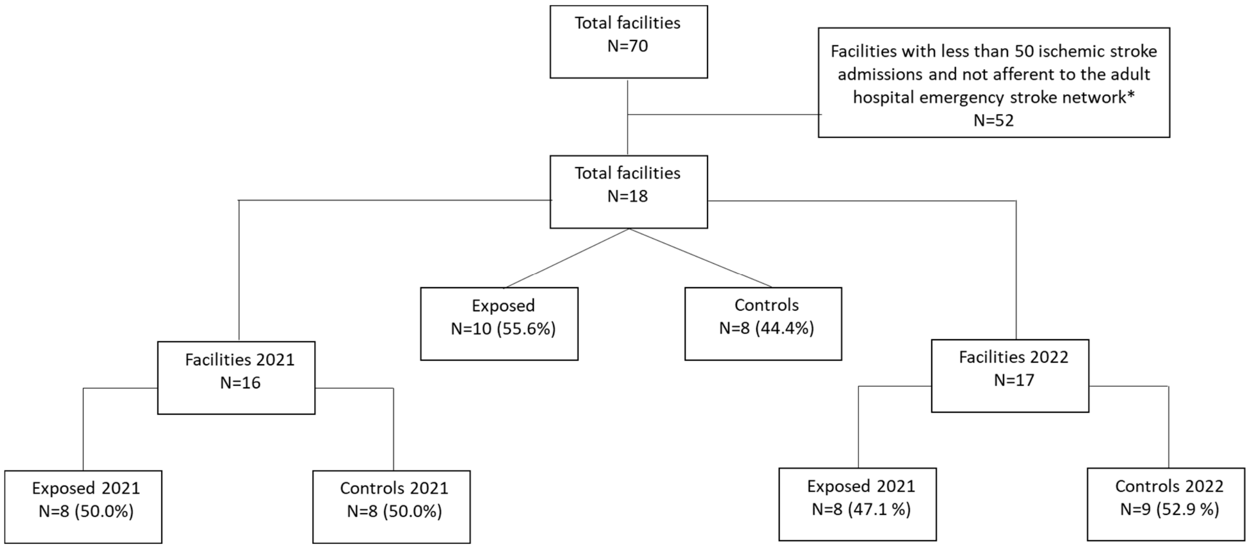

*https://www.regione.lazio.it/enti/salute/sistemi-emergenza/rete-ictus-

Table S5. Distribution of patients belonging to the IMA cohorts by year of analysis and facility.

FACILITIES	PRE-2021	POST-2022	Total
	N (col%)	N (col%)	N (col%)
	**5986 (49.1)**	**6210 (50.9)**	**12196 (100)**
**EXPOSED**	**3393 (48.5)**	**3609 (51.5)**	**7002 (100)**
A.O. S.CAMILLO FORLANINI	289 (8.5)	321 (8.9)	610 (8.7)
A.O.U.U. S.ANDREA	354 (10.4)	345 (9.6)	699 (10.0)
NUOVO OSPEDALE DEI CASTELLI	_	111 (3.1)	111 (1.6)
OSP. DI BELCOLLE	386 (11.4)	366 (10.1)	752 (10.7)
OSP. F.SPAZIANI	301 (8.9)	310 (8.6)	611 (8.7)
OSP. G.GRASSI	261 (7.9)	256 (7.1)	517 (7.4)
OSP. L.PARODI DELFINO	70 (2.1)	61 (1.7)	131 (1.9)
OSP. S.CAMILLO DE LELLIS	260 (7.7)	208 (5.8)	468 (6.7)
OSP. S.EUGENIO	182 (5.4)	178 (4.9)	360 (5.1)
OSP. S.MARIA GORETTI	419 (12.4)	448 (12.4)	867 (12.4)
OSP. S.PAOLO	40 (1.2)	51 (1.4)	91 (1.3)
OSP. S.PERTINI	232 (6.8)	260 (7.2)	492 (7.0)
OSP. S.SPIRITO	177 (5.3)	210 (5.8)	387 (5.5)
OSP.C. G.VANNINI	102 (3.1)	204 (5.7)	306 (4.4)
POL.U. A.GEMELLI	320 (9.4)	280 (7.8)	600 (8.6)
**CONTROLS**	**2593 (49.9)**	**2601 (50.1)**	**5194 (100)**
A.O. S.GIOVANNI ADDOLORATA	223 (8.6)	208 (8.0)	431 (8.3)
A.O.U.U. TOR VERGATA	385 (14.85)	344 (13.2)	729 (14.0)
A.O.U.U. UMBERTO I	184 (7.1)	168 (6.5)	352 (6.8)
C.C.A. AURELIA HOSPITAL	202 (7.79)	168 (6.5)	370 (7.1)
C.C.A.S.ANNA	101 (3.9)	86 (3.3)	187 (3.6)
OSP. DI ANZIO E NETTUNO	48 (1.85)	33 (1.3)	81 (1.6)
OSP. DONO SVIZZERO	245 (9.45)	257 (9.9)	502 (9.7)
OSP. S.GIOVANNI EVENGELISTA	155 (5.98)	188 (7.2)	343 (6.6)
OSP. S.SCOLASTICA	43 (1.66)	51 (2.0)	94 (1.8)
OSP. S.SEBASTIANO	99 (3.82)	61 (2.4)	160 (3.1)
OSP. SS.TRINITA'	61 (2.35)	60 (2.1)	121 (2.3)
OSP.C. S.PIETRO FATEBENEFRATELLI	176 (6.79)	238 (9.2)	414 (8.0)
OSPEDALE FATEBENEFRATELLI-ISOLA TIBERINA	_	78 (3.0)	78 (1.5)
POL.U. CAMPUS BIOMEDICO	66 (2.55)	103 (3.7)	169 (3.3)
POLICLINICO CASILINO	342 (13.19)	300 (11.3)	642 (12.4)
PRESIDIO OSPEDALIERO SAN FILIPPO NERI	263 (10.14)	258 (9.2)	521 (10.0)

Table S6. Distribution of patients belonging to the STEMI cohorts by year of analysis and facility.

FACILITIES	PRE-2021	POST-2022	Totale
	**N(col%)**	**N(col%)**	**N(col%)**
	**2433 (47.8)**	**2651 (52.5)**	**5084 (100)**
**EXPOSED**	**1541 (47.1)**	**1731 (52.9)**	**3272 (100)**
A.O. S.CAMILLO FORLANINI	136 (8.8)	160 (9.2)	296 (9.1)
A.O.U.U. S.ANDREA	164 (10.6)	172 (9.9)	336 (10.3)
OSP. DI BELCOLLE	161 (10.5)	166 (9.6)	327 (10.0)
OSP. F.SPAZIANI	206 (13.4)	198 (11.4)	404 (12.4)
OSP. G.GRASSI	110 (7.1)	109 (6.3)	219 (6.7)
OSP. S.CAMILLO DE LELLIS	71 (4.6)	80 (4.6)	151 (4.6)
OSP. S.EUGENIO	108 (7.0)	108 (6.2)	216 (6.6)
OSP. S.MARIA GORETTI	201 (13.0)	249 (14.4)	450 (13.8)
OSP. S.PERTINI	122 (7.9)	143 (8.3)	265 (8.1)
OSP. S.SPIRITO	55 (3.6)	76 (4.4)	131 (4.0)
OSP.C. G.VANNINI	39 (2.5)	81 (4.7)	120 (3.7)
POL.U. A.GEMELLI	168 (10.9)	189 (10.9)	357 (10.9)
**CONTROLS**	**892 (49.2)**	**920 (50.8)**	**1812 (100)**
A.O. S.GIOVANNI ADDOLORATA	82 (9.2)	89 (9.7)	171 (9.4)
A.O.U.U. TOR VERGATA	224 (25.1)	250 (27.2)	474 (26.2)
A.O.U.U. UMBERTO I	124 (13.9)	99 (10.7)	223 (12.3)
OSP. DONO SVIZZERO	94 (10.5)	117 (12.7)	211 (11.6)
OSP. S.GIOVANNI EVENGELISTA	82 (9.2)	87 (9.5)	169 (9.3)
OSP.C. S.PIETRO FATEBENEFRATELLI	58 (6.5)	67 (7.8)	125 (6.9)
POLICLINICO CASILINO	168 (18.8)	155 (16.9)	323 (17.8)
PRESIDIO OSPEDALIERO SAN FILIPPO NERI	60 (6.7)	56 (6.1)	116 (6.4)

Table S7. Distribution of patients belonging to stroke cohorts by year of analysis and facility (a and b).

a) Intrahospital mortality within 30-days after first hospital admission for ischemic stroke.

FACILITIES	PRE-2021	PRE-2022	Totale
	N(col%)	N(col%)	N(col%)
	**2954 (49.7)**	**2995 (50.3)**	**5949 (100)**
**EXPOSED**	**1885 (49.7)**	**1908 (50.3)**	**3793 (100)**
A.O.U.U. S.ANDREA	206 (10.9)	200 (10.5)	406 (10.7)
A.O.U.U. TOR VERGATA	448 (23.8)	419 (22.0)	867 (22.9)
A.O.U.U. UMBERTO I	308 (16.3)	350 (18.3)	658 (17.4)
OSP. DI BELCOLLE	167 (8.9)	191 (10.0)	358 (9.5)
OSP. S.CAMILLO DE LELLIS	88 (4.7)	89 (4.7)	177 (4.7)
OSP. S.EUGENIO	167 (8.9)	118 (6.2)	285 (7.5)
OSP. S.MARIA GORETTI	149 (7.9)	225 (11.8)	374 (9.9)
POL.U. A.GEMELLI	352 (18.7)	316 (16.6)	668 (17.6)
**CONTROLS**	**1069 (49.6)**	**1087 (50.4)**	**2156 (100)**
A.O. S.CAMILLO FORLANINI	260 (24.3)	245 (22.5)	505 (23.4)
A.O. S.GIOVANNI ADDOLORATA	278 (26.0)	226 (20.8)	504 (23.4)
NUOVO OSPEDALE DEI CASTELLI	_	56 (5.2)	56 (2.6)
OSP. DONO SVIZZERO	46 (4.3)	48 (4.4)	94 (4.4)
OSP. F.SPAZIANI	107 (10.0)	116 (10.7)	223 (10.3)
OSP. SS.TRINITA'	_	79 (7.3)	79 (3.7)
OSP.C. S.PIETRO FATEBENEFRATELLI	46 (4.3)	_	46 (2.1)
POL.U. CAMPUS BIOMEDICO	208 (19.5)	198 (18.2)	406 (18.8)
POLICLINICO CASILINO	45 (4.2)	36 (3.3)	81 (3.8)
PRESIDIO OSPEDALIERO SAN FILIPPO NERI	79 (7.4)	83 (7.6)	162 (7.5)

b) Hospital readmissions within 30-days after discharge for ischemic stroke.

FACILITIES	PRE-2021	PRE-2022	Totale
	**2685 (49.2)**	**2768 (50.8)**	**5453(100)**
**EXPOSED**	**1711 (49.3)**	**1760 (50.7)**	**3471 (100)**
A.O.U.U. S.ANDREA	186 (10.9)	182 (10.4)	368 (10.6)
A.O.U.U. TOR VERGATA	424 (24.8)	398 (22.6)	822 (23.7)
A.O.U.U. UMBERTO I	271 (15.8)	315 (17.9)	586 (16.9)
OSP. DI BELCOLLE	155 (9.1)	184 (10.5)	339 (9.8)
OSP. S.CAMILLO DE LELLIS	82 (4.8)	76 (4.3)	158 (4.6)
OSP. S.EUGENIO	154 (9.0)	108 (6.1)	262 (7.6)
OSP. S.MARIA GORETTI	129 (7.5)	197 (11.2)	326 (9.4)
POL.U. A.GEMELLI	310 (18.2)	300 (17.1)	610 (17.6)
**CONTROLS**	**974 (49.1)**	**1008 (50.9)**	**1982 (100)**
A.O. S.CAMILLO FORLANINI	236 (24.3)	224 (22.2)	460 (23.2)
A.O. S.GIOVANNI ADDOLORATA	245 (25.2)	204 (20.2)	449 (22.7)
NUOVO OSPEDALE DEI CASTELLI	_	54 (5.4)	54 (2.7)
OSP. DONO SVIZZERO	39 (4.0)	48 (4.8)	87 (4.4)
OSP. F.SPAZIANI	98 (10.1)	106 (10.5)	204 (10.3)
OSP. SS.TRINITA'	_	64 (6.4)	64 (3.2)
OSP.C. S.PIETRO FATEBENEFRATELLI	46 (4.7)	_	46 (2.3)
POL.U. CAMPUS BIOMEDICO	201 (20.6)	193 (19.2)	394 (19.9)
POLICLINICO CASILINO	40 (4.1)	35 (3.5)	75 (3.8)
PRESIDIO OSPEDALIERO SAN FILIPPO NERI	69 (7.1)	80 (7.9)	149 (7.5)

Description of patient populations

AMI patient cohort

Table S8. Characteristics of patients included in the AMI cohort in 2021 and 2022 from participating facilities according to A&F intervention exposure status.

	Exposed			Control			Total		χ2
	**N**	**col%**	**row%**	**N**	**col%**	**row%**	**N**	**col%**	**p-value**
**Total**	**7002**			**5194**			**12196**		
**Sex**									0.073
Female	2025	28.9	56.2	1580	30.4	43.8	3605	29.6	
Male	4977	71.1	57.9	3614	69.6	42.1	8591	70.4	
**Age (years)**									0.959
19-59	1778	25.4	57.5	1315	25.3	42.5	3093	25.4	
60-69	1760	25.1	57.4	1308	25.2	42.6	3068	25.2	
70-79	1741	24.9	57.7	1274	24.5	42.3	3015	24.7	
80-100	1723	24.6	57.1	1297	25.0	42.9	3020	24.8	
**Education level**									0.971
Degree	724	10.3	58.2	520	10.0	41.8	1244	10.2	
Lower middle high school	2269	32.4	57.5	1677	32.3	42.5	3946	32.4	
Middle high school	1989	28.4	57.4	1479	28.5	42.6	3468	28.4	
None or elementary	1729	24.7	57.0	1303	25.1	43.0	3032	24.9	
Not stated	291	4.2	57.5	215	4.1	42.5	506	4.2	
**Concomitant clinical conditions**									
Cancer	383	5.5	56.8	291	5.6	43.2	674	5.5	0.751
Diabetes	400	5.7	55.4	322	6.2	44.6	722	5.9	0.260
Lipid metabolism disorders	187	2.7	52.8	167	3.2	47.2	354	2.9	0.077
Obesity	34	0.5	51.5	32	0.6	48.5	66	0.5	0.331
Obesity at index admission	324	4.6	60.2	214	4.1	39.8	538	4.4	0.178
Anaemia	173	2.5	53.1	153	3.0	46.9	326	2.7	0.108
Anaemia at index admission	323	4.6	57.9	235	4.5	42.1	558	4.6	0.817
Coagulation defects	4	0.1	66.7	2	0.0	33.3	6	0.1	0.647
Coagulation defects at index admission	2	0.0	66.7	1	0.0	33.3	3	0.0	0.746
Other haematological diseases	15	0.2	48.4	16	0.3	51.6	31	0.3	0.309
Other haematological diseases at index admission	28	0.4	60.9	18	0.4	39.1	46	0.4	0.635
Arterial hypertension	666	9.5	53.8	571	11.0	46.2	1237	10.1	0.007
Previous myocardial infarction	547	7.8	57.6	402	7.7	42.4	949	7.8	0.883
Other forms of ischemic heart disease	567	8.1	57.0	428	8.2	43.0	995	8.2	0.776
Heart failure	300	4.3	55.4	242	4.7	44.6	542	4.4	0.321
Not well-defined forms and complications of heart disease	41	0.6	54.7	34	0.7	45.3	75	0.6	0.630
Rheumatic heart disease	24	0.3	55.8	19	0.4	44.2	43	0.4	0.832
Rheumatic heart disease at index admission	98	1.4	61.3	62	1.2	38.8	160	1.3	0.323
Cardiomyopathies	37	0.5	67.3	18	0.4	32.7	55	0.5	0.138
Cardiomyopathies at index admission	72	1.0	51.8	67	1.3	48.2	139	1.1	0.178
Acute endocarditis and myocarditis	5	0.1	83.3	1	0.0	16.7	6	0.1	0.199
Other cardiac conditions	61	0.9	58.1	44	0.9	41.9	105	0.9	0.887
Other cardiac conditions at index admission	186	2.7	57.4	138	2.7	42.6	324	2.7	0.999
Conduction disorders and arrhythmias	312	4.5	54.4	262	5.0	45.6	574	4.7	0.129
Cerebrovascular diseases	233	3.3	56.4	180	3.5	43.6	413	3.4	0.677
Cerebrovascular diseases at index admission	183	2.6	54.8	151	2.9	45.2	334	2.7	0.326
Vascular diseases	169	2.4	56.3	131	2.5	43.7	300	2.5	0.702
Vascular diseases at index admission	213	3.0	57.3	159	3.1	42.7	372	3.1	0.951
Chronic obstructive pulmonary disease (COPD)	149	2.1	51.9	138	2.7	48.1	287	2.4	0.057
Chronic nephropathy	252	3.6	58.3	180	3.5	41.7	432	3.5	0.693
Chronic kidney disease	567	8.1	53.4	494	9.5	46.6	1061	8.7	0.006
Chronic diseases (liver, pancreas, intestines)	46	0.7	67.6	22	0.4	32.4	68	0.6	0.087
Chronic diseases (liver, pancreas, intestine) at index admission	46	0.7	65.7	24	0.5	34.3	70	0.6	0.159
Previous coronary artery bypass grafting	202	2.9	63.5	116	2.2	36.5	318	2.6	0.026
Previous coronary angioplasty	757	10.8	61.4	476	9.2	38.6	1233	10.1	0.003
Cerebrovascular revascularization	39	0.6	51.3	37	0.7	48.7	76	0.6	0.281
Other heart surgery	49	0.7	66.2	25	0.5	33.8	74	0.6	0.125
Other vessel surgery	154	2.2	58.6	109	2.1	41.4	263	2.2	0.705
**Type of hospital***									<.0001
EADI	4794	68.5	59.0	3335	64.2	41.0	8129	66.7	
EADII	2077	29.7	57.9	1512	29.1	42.1	3589	29.4	
ER	131	1.9	27.4	347	6.7	72.6	478	3.9	

**EADI: Emergency Admission Department level I;*
EADII: Emergency Admission Department level II;
ER: Emergency Room.

Table S9. Characteristics of patients included in the AMI cohort in 2021 (PRE) from participating facilities by 30-days mortality after first hospital admission in patients with AMI (Yes/No).

	30-days mortality after first hospital admission in patients with AMI								χ2 p-value
	Yes			No			Total		
	N	col%	row %	N	col%	row %	N	col%	
**Total**	**409**			**5577**			**5986**		
**A&F intervention**									0.048
Exposed	251	61.4	7.4	3142	56.3	92.6	3393	56.7	
Control	158	38.6	6.1	2435	43.7	93.9	2593	43.3	
**Sex**									<.0001
Female	169	41.3	9.5	1610	28.9	90.5	1779	29.7	
Male	240	58.7	5.7	3967	71.1	94.3	4207	70.3	
**Age (years)**									<.0001
19-59	31	7.6	2.0	1507	27.0	98.0	1538	25.7	
60-69	45	11.0	3.0	1461	26.2	97.0	1506	25.2	
70-79	107	26.2	7.2	1379	24.7	92.8	1486	24.8	
80-100	226	55.3	15.5	1230	22.1	84.5	1456	24.3	
**Education level**									<.0001
Bachelor's degree	34	8.3	5.9	547	9.8	94.1	581	9.7	
Lower middle high school	102	24.9	5.3	1838	33.0	94.7	1940	32.4	
Middle high school	86	21.0	5.1	1611	28.9	94.9	1697	28.3	
None or elementary	172	42.1	11.1	1375	24.7	88.9	1547	25.8	
Not stated	15	3.7	6.8	206	3.7	93.2	221	3.7	
**Concomitant clinical conditions**									
Cancer	42	10.3	13.0	282	5.1	87.0	324	5.4	<.0001
Diabetes	42	10.3	12.5	295	5.3	87.5	337	5.6	<.0001
Lipid metabolism disorders	12	2.9	7.3	152	2.7	92.7	164	2.7	0.803
Obesity	3	0.7	10.3	26	0.5	89.7	29	0.5	0.452
Obesity at index admission	7	1.7	2.6	262	4.7	97.4	269	4.5	0.005
Anaemias	34	8.3	21.5	124	2.2	78.5	158	2.6	<.0001
Anaemias at index admission	27	6.6	10.5	229	4.1	89.5	256	4.3	0.016
Coagulation defects	_	_	_	3	0.1	100.0	3	0.1	0.639
Coagulation defects at index admission	_	_	_	1	0.0	100.0	1	0.0	0.787
Other haematological diseases	1	0.2	5.0	19	0.3	95.0	20	0.3	0.745
Other haematological diseases at index admission	2	0.5	7.7	24	0.4	92.3	26	0.4	0.862
Arterial hypertension	52	12.7	8.5	558	10.0	91.5	610	10.2	0.081
Previous myocardial infarction	29	7.1	5.9	462	8.3	94.1	491	8.2	0.396
Other forms of ischemic heart disease	51	12.5	10.1	452	8.1	89.9	503	8.4	0.002
Heart failure	48	11.7	17.9	220	3.9	82.1	268	4.5	<.0001
Not well-defined forms and complications of heart disease	4	1.0	8.7	42	0.8	91.3	46	0.8	0.615
Rheumatic heart disease	3	0.7	11.1	24	0.4	88.9	27	0.5	0.377
Rheumatic heart disease at index admission	18	4.4	20.5	70	1.3	79.5	88	1.5	<.0001
Cardiomyopathies	6	1.5	20.0	24	0.4	80.0	30	0.5	0.004
Cardiomyopathies at index admission	7	1.7	10.1	62	1.1	89.9	69	1.2	0.273
Acute endocarditis and myocarditis	_	_	_	5	0.1	100.0	5	0.1	0.545
Other cardiac conditions	7	1.7	15.2	39	0.7	84.8	46	0.8	0.024
Other cardiac conditions at index admission	22	5.4	11.9	163	2.9	88.1	185	3.1	0.006
Conduction disorders and arrhythmias	39	9.5	13.1	258	4.6	86.9	297	5.0	<.0001
Cerebrovascular diseases	23	5.6	11.9	171	3.1	88.1	194	3.2	0.005
Cerebrovascular diseases at index admission	18	4.4	11.3	141	2.5	88.7	159	2.7	0.023
Vascular diseases	21	5.1	14.6	123	2.2	85.4	144	2.4	0.000
Vascular diseases at index admission	12	2.9	7.0	159	2.9	93.0	171	2.9	0.923
Chronic obstructive pulmonary disease (COPD)	18	4.4	12.0	132	2.4	88.0	150	2.5	0.011
Chronic nephropathy	32	7.8	15.2	178	3.2	84.8	210	3.5	<.0001
Chronic kidney disease	59	14.4	11.7	447	8.0	88.3	506	8.5	<.0001
Chronic diseases (liver, pancreas, intestines)	6	1.5	15.4	33	0.6	84.6	39	0.7	0.034
Chronic diseases (liver, pancreas, intestine) at index admission	3	0.7	8.8	31	0.6	91.2	34	0.6	0.645
Previous coronary artery bypass grafting	19	4.7	11.8	142	2.6	88.2	161	2.7	0.011
Previous coronary angioplasty	31	7.6	4.9	607	10.9	95.1	638	10.7	0.037
Cerebrovascular revascularization	4	1.0	13.8	25	0.5	86.2	29	0.5	0.136
Other heart surgery	7	1.7	21.2	26	0.5	78.8	33	0.6	0.001
Other vessel surgery	19	4.7	14.7	110	2.0	85.3	129	2.2	0.000
**Type of hospital***									0.028
EADI	267	65.3	6.9	3629	65.1	93.1	3896	65.1	
EADII	134	32.8	7.4	1686	30.2	92.6	1820	30.4	
ER	8	2.0	3.0	262	4.7	97.0	270	4.5	

**EADI: Emergency Admission Department level I;*
EADII: Emergency Admission Department level II;
ER: Emergency Room.

Table S10. Characteristics of patients included in the AMI cohort in 2022 (POST) from participating facilities by 30-days mortality after first hospital admission in patients with AMI (Yes/No).

	30-days mortality after first hospital admission in patients with AMI	χ2 p-value

	Yes			No			Total		
	N	col%	row %	N	col%	row %	N	col%	
**Total**	**430**			**5780**			**6210**		
**A&F intervention**									0.103
Exposed	266	61.9	7.4	3343	57.8	92.6	3609	58.1	
Control	164	38.1	6.3	2437	42.2	93.7	2601	41.9	
**Sex**								0.0	<.0001
Female	185	43.0	10.1	1641	28.4	89.9	1826	29.4	
Male	245	57.0	5.6	4139	71.6	94.4	4384	70.6	
**Age (years)**								0.0	
19-59	29	6.7	1.9	1526	26.4	98.1	1555	25.0	<.0001
60-69	47	10.9	3.0	1515	26.2	97.0	1562	25.2	
70-79	113	26.3	7.4	1416	24.5	92.6	1529	24.6	
80-100	241	56.1	15.4	1323	22.9	84.6	1564	25.2	
**Education level**								0.0	<.0001
Bachelor's degree	35	8.1	5.3	628	10.9	94.7	663	10.7	
Lower middle high school	113	26.3	5.6	1893	32.8	94.4	2006	32.3	
Middle high school	90	20.9	5.1	1681	29.1	94.9	1771	28.5	
None or elementary	168	39.1	11.3	1317	22.8	88.7	1485	23.9	
Not stated	24	5.6	8.4	261	4.5	91.6	285	4.6	
**Concomitant clinical conditions**								0.0	
Cancer	55	12.8	15.7	295	5.1	84.3	350	5.6	<.0001
Diabetes	47	10.9	12.2	338	5.9	87.8	385	6.2	<.0001
Lipid metabolism disorders	13	3.0	6.8	177	3.1	93.2	190	3.1	0.964
Obesity	2	0.5	5.4	35	0.6	94.6	37	0.6	0.715
Obesity at index admission	6	1.4	2.2	263	4.6	97.8	269	4.3	0.002
Anaemias	28	6.5	16.7	140	2.4	83.3	168	2.7	<.0001
Anaemias at index admission	27	6.3	8.9	275	4.8	91.1	302	4.9	0.157
Coagulation defects	1	0.2	33.3	2	0.0	66.7	3	0.0	0.072
Coagulation defects at index admission	_	_	_	2	0.0	100.0	2	0.0	0.700
Other haematological diseases	1	0.2	9.1	10	0.2	90.9	11	0.2	0.777
Other haematological diseases at index admission	2	0.5	10.0	18	0.3	90.0	20	0.3	0.587
Arterial hypertension	60	14.0	9.6	567	9.8	90.4	627	10.1	0.006
Previous myocardial infarction	25	5.8	5.5	433	7.5	94.5	458	7.4	0.199
Other forms of ischemic heart disease	39	9.1	7.9	453	7.8	92.1	492	7.9	0.361
Heart failure	42	9.8	15.3	232	4.0	84.7	274	4.4	<.0001
Not well-defined forms and complications of heart disease	2	0.5	6.9	27	0.5	93.1	29	0.5	0.995
Rheumatic heart disease	3	0.7	18.8	13	0.2	81.3	16	0.3	0.062
Rheumatic heart disease at index admission	9	2.1	12.5	63	1.1	87.5	72	1.2	0.061
Cardiomyopathies	2	0.5	8.0	23	0.4	92.0	25	0.4	0.832
Cardiomyopathies at index admission	1	0.2	1.4	69	1.2	98.6	70	1.1	0.069
Acute endocarditis and myocarditis	_	_	_	1	0.0	100.0	1	0.0	0.785
Other cardiac conditions	7	1.6	11.9	52	0.9	88.1	59	1.0	0.133
Other cardiac conditions at index admission	13	3.0	9.4	126	2.2	90.6	139	2.2	0.254
Conduction disorders and arrhythmias	36	8.4	13.0	241	4.2	87.0	277	4.5	<.0001
Cerebrovascular diseases	24	5.6	11.0	195	3.4	89.0	219	3.5	0.017
Cerebrovascular diseases at index admission	16	3.7	9.1	159	2.8	90.9	175	2.8	0.241
Vascular diseases	19	4.4	12.2	137	2.4	87.8	156	2.5	0.009
Vascular diseases at index admission	12	2.8	6.0	189	3.3	94.0	201	3.2	0.588
Chronic obstructive pulmonary disease (COPD)	20	4.7	14.6	117	2.0	85.4	137	2.2	0.000
Chronic nephropathy	36	8.4	16.2	186	3.2	83.8	222	3.6	<.0001
Chronic kidney disease	57	13.3	10.3	498	8.6	89.7	555	8.9	0.001
Chronic diseases (liver, pancreas, intestines)	3	0.7	10.3	26	0.5	89.7	29	0.5	0.467
Chronic diseases (liver, pancreas, intestine) at index admission	4	0.9	11.1	32	0.6	88.9	36	0.6	0.321
Previous coronary artery bypass grafting	12	2.8	7.6	145	2.5	92.4	157	2.5	0.719
Previous coronary angioplasty	34	7.9	5.7	561	9.7	94.3	595	9.6	0.221
Cerebrovascular revascularization	2	0.5	4.3	45	0.8	95.7	47	0.8	0.469
Other heart surgery	6	1.4	14.6	35	0.6	85.4	41	0.7	0.051
Other vessel surgery	11	2.6	8.2	123	2.1	91.8	134	2.2	0.554
**Type of hospital***								0.0	0.807
EADI	287	66.7	6.8	3946	68.3	93.2	4233	68.2	
EADII	128	29.8	7.2	1641	28.4	92.8	1769	28.5	
ER	15	3.5	7.2	193	3.3	92.8	208	3.3	

**EADI: Emergency Admission Department level I;*
EADII: Emergency Admission Department level II;
ER: Emergency Room.

STEMI patient cohort

Table S11. Characteristics of patients included in the STEMI cohort in 2021 and 2022 from participating facilities according to A&F intervention exposure status.

	Exposed			Control			Total		χ2
	N	col%	row%	N	col%	row%	N	col%	p-value
**Total**	**3272**			**1812**			**5084**		
**Sex**									0.408
Female	816	24.9	63.4	471	26.0	36.6	1287	25.3	
Male	2456	75.1	64.7	1341	74.0	35.3	3797	74.7	
**Age (years)**									0.688
21-57	825	25.2	63.1	482	26.6	36.9	1307	25.7	
58-65	782	23.9	64.4	433	23.9	35.6	1215	23.9	
66-75	840	25.7	64.6	460	25.4	35.4	1300	25.6	
76-100	825	25.2	65.4	437	24.1	34.6	1262	24.8	
**Education level**									0.355
Degree	368	11.3	63.8	209	11.5	36.2	577	11.4	
Lower middle high school	1073	32.8	64.8	584	32.2	35.2	1657	32.6	
Middle high school	1022	31.2	65.1	547	30.2	34.9	1569	30.9	
None or elementary	653	20.0	64.3	362	20.0	35.7	1015	20.0	
Not stated	156	4.8	58.6	110	6.1	41.4	266	5.2	
**Concomitant clinical conditions**									
Cancer	154	4.7	63.4	89	4.9	36.6	243	4.8	0.743
Diabetes	99	3.0	61.5	62	3.4	38.5	161	3.2	0.440
Lipid metabolism disorders	48	1.5	60.0	32	1.8	40.0	80	1.6	0.412
Obesity	12	0.4	75.0	4	0.2	25.0	16	0.3	0.373
Obesity at index admission	161	4.9	72.5	61	3.4	27.5	222	4.4	0.009
Anaemias	33	1.0	55.0	27	1.5	45.0	60	1.2	0.128
Anaemias at index admission	97	3.0	61.0	62	3.4	39.0	159	3.1	0.370
Coagulation defects	_	_	_	1	0.1	100.0	1	0.0	0.179
Coagulation defects at index admission	1	0.0	100.0	_	_	_	1	0.0	0.457
Other hematological diseases	10	0.3	66.7	5	0.3	33.3	15	0.3	0.852
Other hematological diseases at index admission	13	0.4	76.5	4	0.2	23.5	17	0.3	0.296
Arterial hypertension	199	6.1	61.2	126	7.0	38.8	325	6.4	0.224
Previous myocardial infarction	152	4.7	72.0	59	3.3	28.0	211	4.2	0.017
Other forms of ischemic heart disease	149	4.6	68.7	68	3.8	31.3	217	4.3	0.176
Heart failure	59	1.8	71.1	24	1.3	28.9	83	1.6	0.197
Not well-defined forms and complications of heart disease	8	0.2	61.5	5	0.3	38.5	13	0.3	0.832
Rheumatic heart disease	5	0.2	62.5	3	0.2	37.5	8	0.2	0.913
Rheumatic heart disease at index admission	32	1.0	74.4	11	0.6	25.6	43	0.9	0.167
Cardiomyopathies	10	0.3	62.5	6	0.3	37.5	16	0.3	0.876
Cardiomyopathie at index admission	25	0.8	43.1	33	1.8	56.9	58	1.1	0.001
Acute endocarditis and myocarditis	1	0.0	100.0	_	_	_	1	0.0	0.457
Other cardiac conditions	8	0.2	61.5	5	0.3	38.5	13	0.3	0.832
Other cardiac conditions at index admission	49	1.5	74.2	17	0.9	25.8	66	1.3	0.092
Conduction disorders and arrhythmias	72	2.2	59.0	50	2.8	41.0	122	2.4	0.212
Cerebrovascular diseases	77	2.4	64.2	43	2.4	35.8	120	2.4	0.965
Cerebrovascular diseases at index admission	76	2.3	62.3	46	2.5	37.7	122	2.4	0.630
Vascular diseases	54	1.7	67.5	26	1.4	32.5	80	1.6	0.554
Vascular diseases at index admission	69	2.1	65.1	37	2.0	34.9	106	2.1	0.873
Chronic obstructive pulmonary disease (COPD)	43	1.3	71.7	17	0.9	28.3	60	1.2	0.235
Chronic nephropathy	69	2.1	71.1	28	1.5	28.9	97	1.9	0.160
Chronic kidney disease	188	5.8	63.5	108	6.0	36.5	296	5.8	0.754
Chronic diseases (liver, pancreas, intestines)	14	0.4	73.7	5	0.3	26.3	19	0.4	0.395
Chronic diseases (liver, pancreas, intestine) at index admission	22	0.7	81.5	5	0.3	18.5	27	0.5	0.063
Previous coronary artery bypass grafting	34	1.0	73.9	12	0.7	26.1	46	0.9	0.174
Previous coronary angioplasty	235	7.2	74.8	79	4.4	25.2	314	6.2	<.0001
Cerebrovascular revascularization	10	0.3	62.5	6	0.3	37.5	16	0.3	0.876
Other heart surgery	13	0.4	61.9	8	0.4	38.1	21	0.4	0.814
Other vessel surgery	45	1.4	68.2	21	1.2	31.8	66	1.3	0.514
**Type of hospital***									<.0001
DEAI	2169	66.3	69.7	944	52.1	30.3	3113	61.2	
DEAII	1103	33.7	56.0	868	47.9	44.0	1971	38.8	

**EADI: Emergency Admission Department level I;*
EADII: Emergency Admission Department level II;

Table S12. Characteristics of patients included in the STEMI cohort in 2021 (PRE) from participating facilities according to performing of PTCA within 90 minutes of admission to the hospital ER (Yes/No).

	**% PTCA STEMI patients within 90 min of admission to the ED**								**χ2 p-value**
	**Yes**			**No**			**Total**		
	**N**	**col%**	**row %**	**N**	**col %**	**row %**	**N**	**col %**	
**Total**	**1420**			**1013**			**2433**		
**A&F intervention**									
Exposed	904	63.7	58.7	637	62.9	41.3	1541	63.3	0.694
Control	516	36.3	57.8	376	37.1	42.2	892	36.7	
**Sex**									
Female	324	22.8	51.8	301	29.7	48.2	625	25.7	0.000
Male	1096	77.2	60.6	712	70.3	39.4	1808	74.3	
**Age (years)**									
21-57	408	28.7	65.6	214	21.1	34.4	622	25.6	<.0001
58-65	373	26.3	63.5	214	21.1	36.5	587	24.1	
**66-75**	352	24.8	56.2	274	27.1	43.8	626	25.7	
76-100	287	20.2	48.0	311	30.7	52.0	598	24.6	
Education level									
Bachelor's degree	147	10.4	57.0	111	11.0	43.0	258	10.6	0.013
Lower middle high school	481	33.9	59.4	329	32.5	40.6	810	33.3	
**Middle high school**	455	32.0	62.2	277	27.3	37.8	732	30.1	
None or elementary	268	18.9	52.3	244	24.1	47.7	512	21.0	
Not stated	69	4.9	57.0	52	5.1	43.0	121	5.0	
**Concomitant clinical conditions**									
Cancer	47	3.3	39.5	72	7.1	60.5	119	4.9	<.0001
Diabetes	28	2.0	38.9	44	4.3	61.1	72	3.0	0.001
Lipid metabolism disorders	16	1.1	48.5	17	1.7	51.5	33	1.4	0.246
Obesity	1	0.1	16.7	5	0.5	83.3	6	0.2	0.038
Obesity at index admission	61	4.3	50.4	60	5.9	49.6	121	5.0	0.069
Anaemias	8	0.6	26.7	22	2.2	73.3	30	1.2	0.000
Anaemias at index admission	24	1.7	30.4	55	5.4	69.6	79	3.2	<.0001
Coagulation defects	–	–	–	–	–	–	–	–	–
Coagulation defects at index admission	–	–	–	–	–	–	–	–	–
Other haematological diseases	3	0.2	30.0	7	0.7	70.0	10	0.4	0.068
Other haematological diseases at index admission	3	0.2	42.9	4	0.4	57.1	7	0.3	0.405
Arterial hypertension	74	5.2	50.3	73	7.2	49.7	147	6.0	0.042
Previous myocardial infarction	39	2.8	38.6	62	6.1	61.4	101	4.2	<.0001
Other forms of ischemic heart disease	36	2.5	36.4	63	6.2	63.6	99	4.1	<.0001
Heart failure	12	0.9	32.4	25	2.5	67.6	37	1.5	0.001
Not well-defined forms and complications of heart disease	2	0.1	28.6	5	0.5	71.4	7	0.3	0.109
Rheumatic heart disease	2	0.1	40.0	3	0.3	60.0	5	0.2	0.404
Rheumatic heart disease at index admission	6	0.4	30.0	14	1.4	70.0	20	0.8	0.010
Cardiomyopathies	1	0.1	25.0	3	0.3	75.0	4	0.2	0.176
Cardiomyopathies at index admission	10	0.7	34.5	19	1.9	65.5	29	1.2	0.009
Acute endocarditis and myocarditis	–	–	–	–	–	–	–	–	–
Other cardiac conditions	1	0.1	16.7	5	0.5	83.3	6	0.2	0.038
Other cardiac conditions at index admission	9	0.6	30.0	21	2.1	70.0	30	1.2	0.002
Conduction disorders and arrhythmias	20	1.4	33.3	40	4.0	66.7	60	2.5	<.0001
Cerebrovascular diseases	20	1.4	36.4	35	3.5	63.6	55	2.3	0.001
Cerebrovascular diseases at index admission	14	1.0	26.9	38	3.8	73.1	52	2.1	<.0001
Vascular diseases	15	1.1	39.5	23	2.3	60.5	38	1.6	0.017
Vascular diseases at index admission	19	1.3	33.9	37	3.7	66.1	56	2.3	0.000
Chronic obstructive pulmonary disease (COPD)	11	0.8	39.3	17	1.7	60.7	28	1.2	0.039
Chronic nephropathy	18	1.3	41.9	25	2.5	58.1	43	1.8	0.027
Chronic kidney disease	63	4.4	45.7	75	7.4	54.3	138	5.7	0.002
Chronic diseases (liver, pancreas, intestines)	5	0.4	50.0	5	0.5	50.0	10	0.4	0.591
Chronic diseases (liver, pancreas, intestine) at index admission	4	0.3	50.0	4	0.4	50.0	8	0.3	0.631
Previous coronary artery bypass grafting	7	0.5	35.0	13	1.3	65.0	20	0.8	0.033
Previous coronary angioplasty	78	5.5	51.7	73	7.2	48.3	151	6.2	0.084
Cerebrovascular revascularization	4	0.3	66.7	2	0.2	33.3	6	0.2	0.680
Other heart surgery	2	0.1	25.0	6	0.6	75.0	8	0.3	0.055
Other vessel surgery	13	0.9	41.9	18	1.8	58.1	31	1.3	0.062
**Type of hospital***								0.0	0.024
EADI	901	63.5	60.1	597	58.9	39.9	1498	61.6	
EADII	519	36.6	55.5	416	41.1	44.5	935	38.4	

**EADI: Emergency Admission Department level I;*
EADII: Emergency Admission Department level II;

Table S13. Characteristics of patients included in the STEMI cohort in 2022 (POST) from participating facilities according to performing of PTCA within 90 minutes of admission to the hospital ER (Yes/No).

	% PTCA STEMI patients within 90 min of admission to the hospital ER								χ2 p-value
	Yes			No			Total		
	N	col%	row %	N	col %	row %	N	col%	
**Total**	**1657**			**994**			**2651**		
**A&F intervention**									
Exposed	1046	63.1	60.4	685	68.9	39.6	1731	65.3	0.002
Control	611	36.9	66.4	309	31.1	33.6	920	34.7	
**Sex**									
Female	377	22.8	56.9	285	28.7	43.1	662	25.0	0.001
Male	1280	77.3	64.4	709	71.3	35.6	1989	75.0	
**Age (years)**									
21-57	471	28.4	68.8	214	21.5	31.2	685	25.8	<.0001
58-65	435	26.3	69.3	193	19.4	30.7	628	23.7	
**66-75**	429	25.9	63.6	245	24.7	36.4	674	25.4	
76-100	322	19.4	48.5	342	34.4	51.5	664	25.0	
Education level									
Bachelor's degree	192	11.6	60.2	127	12.8	39.8	319	12.0	0.002
Lower middle high school	550	33.2	64.9	297	29.9	35.1	847	32.0	
**Middle high school**	533	32.2	63.7	304	30.6	36.3	837	31.6	
None or elementary	280	16.9	55.7	223	22.4	44.3	503	19.0	
Not stated	102	6.2	70.3	43	4.3	29.7	145	5.5	
**Concomitant clinical conditions**									
Cancer	62	3.7	50.0	62	6.2	50.0	124	4.7	0.003
Diabetes	39	2.4	43.8	50	5.0	56.2	89	3.4	0.000
Lipid metabolism disorders	20	1.2	42.6	27	2.7	57.4	47	1.8	0.004
Obesity	6	0.4	60.0	4	0.4	40.0	10	0.4	0.870
Obesity at index admission	65	3.9	64.4	36	3.6	35.6	101	3.8	0.695
Anaemias	8	0.5	26.7	22	2.2	73.3	30	1.1	<.0001
Anaemias at index admission	36	2.2	45.0	44	4.4	55.0	80	3.0	0.001
Coagulation defects	1	0.1	100.0	_	_	_	1	0.0	0.439
Coagulation defects at index admission	1	0.1	100.0	_	_	_	1	0.0	0.439
Other haematological diseases	2	0.1	40.0	3	0.3	60.0	5	0.2	0.298
Other haematological diseases at index admission	8	0.5	80.0	2	0.2	20.0	10	0.4	0.252
Arterial hypertension	92	5.6	51.7	86	8.7	48.3	178	6.7	0.002
Previous myocardial infarction	42	2.5	38.2	68	6.8	61.8	110	4.1	<.0001
Other forms of ischemic heart disease	60	3.6	50.8	58	5.8	49.2	118	4.5	0.008
Heart failure	15	0.9	32.6	31	3.1	67.4	46	1.7	<.0001
Not well-defined forms and complications of heart disease	2	0.1	33.3	4	0.4	66.7	6	0.2	0.140
Rheumatic heart disease	2	0.1	66.7	1	0.1	33.3	3	0.1	0.882
Rheumatic heart disease at index admission	12	0.7	52.2	11	1.1	47.8	23	0.9	0.304
Cardiomyopathies	2	0.1	16.7	10	1.0	83.3	12	0.5	0.001
Cardiomyopathies at index admission	13	0.8	44.8	16	1.6	55.2	29	1.1	0.048
Acute endocarditis and myocarditis	_	_	_	1	0.1	100.0	1	0.0	0.197
Other cardiac conditions	1	0.1	14.3	6	0.6	85.7	7	0.3	0.008
Other cardiac conditions at index admission	12	0.7	33.3	24	2.4	66.7	36	1.4	0.000
Conduction disorders and arrhythmias	25	1.5	40.3	37	3.7	59.7	62	2.3	0.000
Cerebrovascular diseases	34	2.1	52.3	31	3.1	47.7	65	2.5	0.086
Cerebrovascular diseases at index admission	28	1.7	40.0	42	4.2	60.0	70	2.6	<.0001
Vascular diseases	13	0.8	31.0	29	2.9	69.0	42	1.6	<.0001
Vascular diseases at index admission	23	1.4	46.0	27	2.7	54.0	50	1.9	0.015
Chronic obstructive pulmonary disease (COPD)	12	0.7	37.5	20	2.0	62.5	32	1.2	0.003
Chronic nephropathy	19	1.2	35.2	35	3.5	64.8	54	2.0	<.0001
Chronic kidney disease	68	4.1	43.0	90	9.1	57.0	158	6.0	<.0001
Chronic diseases (liver, pancreas, intestines)	4	0.2	44.4	5	0.5	55.6	9	0.3	0.262
Chronic diseases (liver, pancreas, intestine) at index admission	9	0.5	47.4	10	1.0	52.6	19	0.7	0.171
Previous coronary artery bypass grafting	12	0.7	46.2	14	1.4	53.8	26	1.0	0.084
Previous coronary angioplasty	81	4.9	49.7	82	8.3	50.3	163	6.1	0.001
Cerebrovascular revascularization	6	0.4	60.0	4	0.4	40.0	10	0.4	0.870
Other heart surgery	4	0.2	30.8	9	0.9	69.2	13	0.5	0.018
Other vessel surgery	12	0.7	34.3	23	2.3	65.7	35	1.3	0.001
**Type of hospital***									
EADI	1008	60.8	62.4	607	61.1	37.6	1615	60.9	0.905
EADII	649	39.2	62.6	387	38.9	37.4	1036	39.1	

**EADI: Emergency Admission Department level I;*
EADII: Emergency Admission Department level II;

Ischemic Stroke patient cohort

The eligibility criteria for including patients in the stroke cohorts for calculating the two indicators considered are different, so the two cohorts are described separately.

- ✓ In hospital mortality within 30-days of first hospital admission in patients with ischemic stroke

Table S14. Characteristics of patients included in the ischemic stroke cohort (in hospital mortality) in 2021 and 2022 from participating facilities according to A&F intervention exposure status.

	Exposed			Control			Total		χ2
	N	col%	row%	N	col%	row%	N	col%	p-value
**Total**	**3793**			**2156**			**5949**		
**Sex**									0.848
Female	1753	46.2	63.6	1002	46.5	36.4	2755	46.3	
Male	2040	53.8	63.9	1154	53.5	36.1	3194	53.7	
**Age (years)**									0.095
35-66	967	25.5	64.1	541	25.1	35.9	1508	25.3	
67-76	959	25.3	65.1	513	23.8	34.9	1472	24.7	
77-83	925	24.4	64.7	504	23.4	35.3	1429	24.0	
84-100	942	24.8	61.2	598	27.7	38.8	1540	25.9	
**Education level**									<.0001
Bachelor's degree	356	9.4	62.8	211	9.8	37.2	567	9.5	
Lower middle high school	997	26.3	62.4	602	27.9	37.6	1599	26.9	
Middle high school	857	22.6	62.1	523	24.3	37.9	1380	23.2	
None or elementary	1355	35.7	64.2	757	35.1	35.8	2112	35.5	
Not stated	228	6.0	78.4	63	2.9	21.6	291	4.9	
**Concomitant clinical conditions**									
Cancer	129	3.4	60.6	84	3.9	39.4	213	3.6	0.323
Diabetes	188	5.0	65.3	100	4.6	34.7	288	4.8	0.583
Lipid metabolism disorders	63	1.7	57.3	47	2.2	42.7	110	1.8	0.153
Obesity	26	0.7	72.2	10	0.5	27.8	36	0.6	0.289
Obesity at index admission	77	2.0	55.4	62	2.9	44.6	139	2.3	0.038
Anaemias	102	2.7	60.0	68	3.2	40.0	170	2.9	0.301
Anaemias at index admission	67	1.8	37.2	113	5.2	62.8	180	3.0	<.0001
Coagulation defects	1	0.0	33.3	2	0.1	66.7	3	0.1	0.273
Coagulation defects at index admission	2	0.1	16.7	10	0.5	83.3	12	0.2	0.001
Other haematological diseases	10	0.3	66.7	5	0.2	33.3	15	0.3	0.815
Other haematological diseases at index admission	24	0.6	55.8	19	0.9	44.2	43	0.7	0.277
Arterial hypertension	370	9.8	62.6	221	10.3	37.4	591	9.9	0.539
Previous myocardial infarction	69	1.8	54.8	57	2.6	45.2	126	2.1	0.034
Other forms of ischemic heart disease	138	3.6	54.5	115	5.3	45.5	253	4.3	0.002
Heart failure	169	4.5	58.9	118	5.5	41.1	287	4.8	0.078
Not well-defined forms and complications of heart disease	35	0.9	85.4	6	0.3	14.6	41	0.7	0.004
Rheumatic heart disease	27	0.7	60.0	18	0.8	40.0	45	0.8	0.599
Rheumatic heart disease at index admission	26	0.7	43.3	34	1.6	56.7	60	1.0	0.001
Cardiomyopathies	17	0.5	56.7	13	0.6	43.3	30	0.5	0.418
Cardiomyopathies at index admission	20	0.5	55.6	16	0.7	44.4	36	0.6	0.304
Acute endocarditis and myocarditis	1	0.0	33.3	2	0.1	66.7	3	0.1	0.273
Other cardiac conditions	43	1.1	52.4	39	1.8	47.6	82	1.4	0.032
Other cardiac conditions at index admission	136	3.6	59.6	92	4.3	40.4	228	3.8	0.188
Conduction disorders and arrhythmias	229	6.0	60.9	147	6.8	39.1	376	6.3	0.234
Cerebrovascular diseases	254	6.7	60.8	164	7.6	39.2	418	7.0	0.187
Vascular diseases	86	2.3	62.8	51	2.4	37.2	137	2.3	0.808
Vascular diseases at index admission	157	4.1	63.3	91	4.2	36.7	248	4.2	0.880
Chronic obstructive pulmonary disease (COPD)	78	2.1	62.9	46	2.1	37.1	124	2.1	0.841
Chronic nephropathy	95	2.5	59.7	64	3.0	40.3	159	2.7	0.286
Chronic nephropathy at index admission	149	3.9	67.7	71	3.3	32.3	220	3.7	0.212
Chronic kidney disease	25	0.7	71.4	10	0.5	28.6	35	0.6	0.344
Diseases chronic diseases (liver, pancreas, intestine)	28	0.7	66.7	14	0.7	33.3	42	0.7	0.694
Diseases chronic diseases (liver, pancreas, intestine) at index admission	84	2.2	58.3	60	2.8	41.7	144	2.4	0.170
Cerebrovascular revascularization	16	0.4	59.3	11	0.5	40.7	27	0.5	0.626
Other heart surgery	42	1.1	62.7	25	1.2	37.3	67	1.1	0.854
Other vessel surgery	78	2.1	60.0	52	2.4	40.0	130	2.2	0.367
**Type of hospital***									<.0001
noNVT	_	_	_	125	5.8	100.0	125	2.1	
NVT	_	_	_	175	8.1	100.0	175	2.9	
NTUI	1600	42.2	54.2	1351	62.7	45.8	2951	49.6	
NTUII	2193	57.8	81.3	505	23.4	18.7	2698	45.4	

** noNVT: Hospital without Neurovascular Treatment Team;*
NVT: Hospital with a Neurovascular Treatment Team;
NTUI: Neurovascular Treatment Unit level I;
NTUII: Neurovascular Treatment Unit level II.

Table S15. Characteristics of patients included in the ischemic stroke cohort in 2021 (PRE) from participating facilities by in hospital 30-days mortality since first hospital admission in patients with ischemic stroke (Yes/No).

	**In hospital 30-days mortality after admission in patients with ischemic stroke**								**χ2 p-value**
	**Yes**			**No**			**Total**		
	**N**	**col%**	**row%**	**N**	**col%**	**row%**	**N**	**col%**	
**Total**	**232**			**2722**			**2954**		
**A&F intervention**									0.008
Exposed	137	59.1	9.2	1359	49.9	90.8	1496	50.6	
Control	95	41.0	6.5	1363	50.1	93.5	1458	49.4	
**Sex**									0.001
Female	131	56.5	9.6	1234	45.3	90.4	1365	46.2	
Male	101	43.5	6.4	1488	54.7	93.6	1589	53.8	
**Age (years)**									<.0001
35-66	14	6.0	1.9	720	26.5	98.1	734	24.8	
67-76	35	15.1	4.6	724	26.6	95.4	759	25.7	
77-83	54	23.3	7.7	647	23.8	92.3	701	23.7	
84-100	129	55.6	17.0	631	23.2	83.0	760	25.7	
**Education level**									0.000
Bachelor's degree	12	5.2	4.2	276	10.1	95.8	288	9.7	
Lower middle high school	57	24.6	7.5	698	25.6	92.5	755	25.6	
Middle high school	40	17.2	5.7	666	24.5	94.3	706	23.9	
None or elementary	115	49.6	10.6	966	35.5	89.4	1081	36.6	
Not stated	8	3.5	6.5	116	4.3	93.5	124	4.2	
**Concomitant clinical conditions**									
Cancer	9	3.9	8.6	96	3.5	91.4	105	3.6	0.781
Diabetes	14	6.0	10.4	121	4.5	89.6	135	4.6	0.266
Lipid metabolism disorders	2	0.9	3.8	50	1.8	96.2	52	1.8	0.278
Obesity	2	0.9	11.8	15	0.6	88.2	17	0.6	0.548
Obesity at index admission	5	2.2	6.2	76	2.8	93.8	81	2.7	0.569
Anaemias	12	5.2	13.2	79	2.9	86.8	91	3.1	0.055
Anaemias at index admission	5	2.2	6.5	72	2.7	93.5	77	2.6	0.653
Coagulation defects	_	_	_	1	0.0	100.0	1	0.0	0.770
Coagulation defects at index admission	_	_	_	10	0.4	100.0	10	0.3	0.355
Other haematological diseases	_	_	_	5	0.2	100.0	5	0.2	0.514
Other haematological diseases at index admission	4	1.7	20.0	16	0.6	80.0	20	0.7	0.043
Arterial hypertension	39	16.8	13.4	252	9.3	86.6	291	9.9	0.000
Previous myocardial infarction	7	3.0	11.7	53	2.0	88.3	60	2.0	0.267
Other forms of ischemic heart disease	15	6.5	11.3	118	4.3	88.7	133	4.5	0.133
Heart failure	25	10.8	16.6	126	4.6	83.4	151	5.1	<.0001
Not well-defined forms and complications of heart disease	4	1.7	19.0	17	0.6	81.0	21	0.7	0.056
Rheumatic heart disease	5	2.2	25.0	15	0.6	75.0	20	0.7	0.004
Rheumatic heart disease at index admission	_	_	_	29	1.1	100.0	29	1.0	0.114
Cardiomyopathies	2	0.9	12.5	14	0.5	87.5	16	0.5	0.489
Cardiomyopathies at index admission	2	0.9	14.3	12	0.4	85.7	14	0.5	0.370
Acute endocarditis and myocarditis	_	_	_	3	0.1	100.0	3	0.1	0.613
Other cardiac conditions	8	3.5	18.2	36	1.3	81.8	44	1.5	0.010
Other cardiac conditions at index admission	5	2.2	4.0	119	4.4	96.0	124	4.2	0.106
Conduction disorders and arrhythmias	29	12.5	15.3	161	5.9	84.7	190	6.4	<.0001
Cerebrovascular diseases	19	8.2	9.0	191	7.0	91.0	210	7.1	0.505
Vascular diseases	5	2.2	8.5	54	2.0	91.5	59	2.0	0.858
Vascular diseases at index admission	9	3.9	7.5	111	4.1	92.5	120	4.1	0.883
Chronic obstructive pulmonary disease (COPD)	11	4.7	17.2	53	2.0	82.8	64	2.2	0.005
Chronic nephropathy	9	3.9	13.4	58	2.1	86.6	67	2.3	0.086
Chronic nephropathy at index admission	18	7.8	17.0	88	3.2	83.0	106	3.6	0.000
Chronic kidney disease	2	0.9	13.3	13	0.5	86.7	15	0.5	0.429
Diseases chronic diseases (liver, pancreas, intestine)	2	0.9	10.0	18	0.7	90.0	20	0.7	0.720
Diseases chronic diseases (liver, pancreas, intestine) at index admission	6	2.6	7.5	74	2.7	92.5	80	2.7	0.905
Cerebrovascular revascularization	_	_	_	15	0.6	100.0	15	0.5	0.257
Other heart surgery	8	3.5	22.2	28	1.0	77.8	36	1.2	0.001
Other vessel surgery	4	1.7	6.6	57	2.1	93.4	61	2.1	0.704
**Type of hospital***									
noNVT	_	_	_	46	1.7	100.0	46	1.6	0.128
NVT	10	4.3	11.0	81	3.0	89.0	91	3.1	
NTUI	109	47.0	7.5	1340	49.2	92.5	1449	49.1	
NTUII	113	48.7	8.3	1255	46.1	91.7	1368	46.3	

* *noNVT: Hospital without Neurovascular Treatment Team;*
NVT: Hospital with a Neurovascular Treatment Team;
NTUI: Neurovascular Treatment Unit level I;
NTUII: Neurovascular Treatment Unit level II.

Table S16. Characteristics of patients included in the ischemic stroke cohort in 2022 (POST) from participating facilities by in hospital 30-days mortality since first hospital admission in patients with ischemic stroke (Yes/No).

	In hospital 30-days mortality after admission in patients with ischemic stroke								χ2 p-value
	Yes			No			Total		
	N	col%	row%	N	col%	row%	N	col%	
**Total**	**200**			**2795**			**2995**		
**A&F intervention**									0.236
Exposed	112	56.0	7.2	1444	51.7	92.8	1556	52.0	
Control	88	44.0	6.1	1351	48.3	93.9	1439	48.0	
**Sex**									0.005
Female	112	56.0	8.1	1278	45.7	91.9	1390	46.4	
Male	88	44.0	5.5	1517	54.3	94.5	1605	53.6	
**Age (years)**									<.0001
35-66	12	6.0	1.6	762	27.3	98.4	774	25.8	
67-76	36	18.0	5.0	677	24.2	95.0	713	23.8	
77-83	57	28.5	7.8	671	24.0	92.2	728	24.3	
84-100	95	47.5	12.2	685	24.5	87.8	780	26.0	
**Education level**									0.001
Bachelor’s degree	12	6.0	4.3	267	9.6	95.7	279	9.3	
Lower middle high school	50	25.0	5.9	794	28.4	94.1	844	28.2	
Middle high school	36	18.0	5.3	638	22.8	94.7	674	22.5	
None or elementary	96	48.0	9.3	935	33.5	90.7	1031	34.4	
Not stated	6	3.0	3.6	161	5.8	96.4	167	5.6	
**Concomitant clinical conditions**								0.0	
Cancer	12	6.0	11.1	96	3.4	88.9	108	3.6	0.060
Diabetes	20	10.0	13.1	133	4.8	86.9	153	5.1	0.001
Lipid metabolism disorders	5	2.5	8.6	53	1.9	91.4	58	1.9	0.550
Obesity	2	1.0	10.5	17	0.6	89.5	19	0.6	0.500
Obesity at index admission	4	2.0	6.9	54	1.9	93.1	58	1.9	0.946
Anaemias	11	5.5	13.9	68	2.4	86.1	79	2.6	0.009
Anaemias at index admission	6	3.0	5.8	97	3.5	94.2	103	3.4	0.724
Coagulation defects	_	_	_	2	0.1	100.0	2	0.1	0.705
Coagulation defects at index admission	1	0.5	50.0	1	0.0	50.0	2	0.1	0.014
Other haematological diseases	1	0.5	10.0	9	0.3	90.0	10	0.3	0.673
Other haematological diseases at index admission	_	_	_	23	0.8	100.0	23	0.8	0.198
Arterial hypertension	32	16.0	10.7	268	9.6	89.3	300	10.0	0.004
Previous myocardial infarction	7	3.5	10.6	59	2.1	89.4	66	2.2	0.196
Other forms of ischemic heart disease	12	6.0	10.0	108	3.9	90.0	120	4.0	0.137
Heart failure	25	12.5	18.4	111	4.0	81.6	136	4.5	<.0001
Not well-defined forms and complications of heart disease	_	_	_	20	0.7	100.0	20	0.7	0.230
Rheumatic heart disease	3	1.5	12.0	22	0.8	88.0	25	0.8	0.284
Rheumatic heart disease at index admission	2	1.0	6.5	29	1.0	93.5	31	1.0	0.960
Cardiomyopathies	1	0.5	7.1	13	0.5	92.9	14	0.5	0.944
Cardiomyopathies at index admission	1	0.5	4.5	21	0.8	95.5	22	0.7	0.688
Acute endocarditis and myocarditis	_	_	_	_	_	_	_	_	_
Other cardiac conditions	4	2.0	10.5	34	1.2	89.5	38	1.3	0.339
Other cardiac conditions at index admission	3	1.5	2.9	101	3.6	97.1	104	3.5	0.115
Conduction disorders and arrhythmias	26	13.0	14.0	160	5.7	86.0	186	6.2	<.0001
Cerebrovascular diseases	21	10.5	10.1	187	6.7	89.9	208	6.9	0.041
Vascular diseases	6	3.0	7.7	72	2.6	92.3	78	2.6	0.716
Vascular diseases at index admission	6	3.0	4.7	122	4.4	95.3	128	4.3	0.357
Chronic obstructive pulmonary disease (COPD)	10	5.0	16.7	50	1.8	83.3	60	2.0	0.002
Chronic nephropathy	17	8.5	18.5	75	2.7	81.5	92	3.1	<.0001
Chronic nephropathy at index admission	11	5.5	9.6	103	3.7	90.4	114	3.8	0.195
Chronic kidney disease	1	0.5	5.0	19	0.7	95.0	20	0.7	0.763
Diseases chronic diseases (liver, pancreas, intestine)	_	_	_	22	0.8	100.0	22	0.7	0.208
Diseases chronic diseases (liver, pancreas, intestine) at index admission	3	1.5	4.7	61	2.2	95.3	64	2.1	0.519
Cerebrovascular revascularization	_	_	_	12	0.4	100.0	12	0.4	0.353
Other heart surgery	5	2.5	16.1	26	0.9	83.9	31	1.0	0.034
Other vessel surgery	7	3.5	10.1	62	2.2	89.9	69	2.3	0.243
**Type of hospital***	15	7.5	19.0	64	2.3	81.0	79	2.6	<.0001
noNVT									
NVT	1	0.5	1.2	83	3.0	98.8	84	2.8	
NTUI	100	50.0	6.7	1402	50.2	93.3	1502	50.2	
NTUII	84	42.0	6.3	1246	44.6	93.7	1330	44.4	

** noNVT: Hospital without Neurovascular Treatment Team;*
NVT: Hospital with a Neurovascular Treatment Team;
NTUI: Neurovascular Treatment Unit level I;
NTUII: Neurovascular Treatment Unit level II.

✓ Hospital readmissions within 30-days of discharge for ischemic stroke

Table S17. Characteristics of patients included in the ischemic stroke cohort (hospital readmissions) in 2021 and 2022 from participating facilities according to A&F intervention exposure status.

	**Exposed**			**Control**			**Total**		**χ2**
	**N**	**col%**	**row%**	**N**	**col%**	**row%**	**N**	**col%**	**p-value**
**Total**	**3471**			**1982**			**5453**		
**Sex**									0.928
Female	1577	45.4	63.6	903	45.6	36.4	2480	45.5	
Male	1894	54.6	63.7	1079	54.4	36.3	2973	54.5	
**Age (years)**									0.046
35-66	874	25.2	63.9	493	24.9	36.1	1367	25.1	
67-76	872	25.1	65.0	469	23.7	35.0	1341	24.6	
77-83	941	27.1	65.0	507	25.6	35.0	1448	26.6	
84-100	784	22.6	60.4	513	25.9	39.6	1297	23.8	
**Education level**									<.0001
Bachelor's degree	337	9.7	62.5	202	10.2	37.5	539	9.9	
Lower middle high school	919	26.5	62.1	561	28.3	37.9	1480	27.1	
Middle high school	800	23.1	61.9	492	24.8	38.1	1292	23.7	
None or elementary	1201	34.6	64.3	667	33.7	35.7	1868	34.3	
Not stated	214	6.2	78.1	60	3.0	21.9	274	5.0	
**Concomitant clinical conditions**									
Cancer	113	3.3	59.2	78	3.9	40.8	191	3.5	0.189
Diabetes	160	4.6	64.0	90	4.5	36.0	250	4.6	0.907
Lipid metabolism disorders	59	1.7	59.0	41	2.1	41.0	100	1.8	0.329
Obesity	23	0.7	71.9	9	0.5	28.1	32	0.6	0.332
Obesity at index admission	73	2.1	56.6	56	2.8	43.4	129	2.4	0.091
Anaemias	89	2.6	61.4	56	2.8	38.6	145	2.7	0.564
Anaemias at index admission	60	1.7	37.3	101	5.1	62.7	161	3.0	<.0001
Coagulation defects	1	0.0	33.3	2	0.1	66.7	3	0.1	0.275
Coagulation defects at index admission	_	_	_	10	0.5	100.0	10	0.2	<.0001
Other haematological diseases	9	0.3	64.3	5	0.3	35.7	14	0.3	0.961
Other haematological diseases at index admission	21	0.6	55.3	17	0.9	44.7	38	0.7	0.281
Arterial hypertension	322	9.3	62.4	194	9.8	37.6	516	9.5	0.535
Previous myocardial infarction	60	1.7	54.1	51	2.6	45.9	111	2.0	0.034
Other forms of ischemic heart disease	122	3.5	55.0	100	5.1	45.0	222	4.1	0.006
Heart failure	132	3.8	56.4	102	5.2	43.6	234	4.3	0.019
Not well-defined forms and complications of heart disease	31	0.9	83.8	6	0.3	16.2	37	0.7	0.011
Rheumatic heart disease	21	0.6	58.3	15	0.8	41.7	36	0.7	0.506
Rheumatic heart disease at index admission	24	0.7	42.1	33	1.7	57.9	57	1.0	0.001
Cardiomyopathies	14	0.4	51.9	13	0.7	48.1	27	0.5	0.201
Cardiomyopathies at index admission	19	0.6	57.6	14	0.7	42.4	33	0.6	0.467
Acute endocarditis and myocarditis	1	0.0	33.3	2	0.1	66.7	3	0.1	0.275
Other cardiac conditions	34	1.0	50.0	34	1.7	50.0	68	1.2	0.019
Other cardiac conditions at index admission	129	3.7	58.9	90	4.5	41.1	219	4.0	0.136
Conduction disorders and arrhythmias	192	5.5	60.8	124	6.3	39.2	316	5.8	0.271
Cerebrovascular diseases	224	6.5	60.2	148	7.5	39.8	372	6.8	0.153
Vascular diseases	77	2.2	61.6	48	2.4	38.4	125	2.3	0.629
Vascular diseases at index admission	143	4.1	61.6	89	4.5	38.4	232	4.3	0.514
Chronic obstructive pulmonary disease (COPD)	67	1.9	65.0	36	1.8	35.0	103	1.9	0.766
Chronic nephropathy	77	2.2	58.8	54	2.7	41.2	131	2.4	0.240
Chronic nephropathy at index admission	129	3.7	68.3	60	3.0	31.7	189	3.5	0.181
Chronic kidney disease	23	0.7	71.9	9	0.5	28.1	32	0.6	0.332
Diseases chronic diseases (liver, pancreas, intestine)	25	0.7	64.1	14	0.7	35.9	39	0.7	0.953
Diseases chronic diseases (liver, pancreas, intestine) at index admission	77	2.2	58.3	55	2.8	41.7	132	2.4	0.198
Cerebrovascular revascularization	16	0.5	59.3	11	0.6	40.7	27	0.5	0.634
Other heart surgery	32	0.9	60.4	21	1.1	39.6	53	1.0	0.618
Other vessel surgery	67	1.9	57.8	49	2.5	42.2	116	2.1	0.182
**Type of hospital***									<.0001
noNVT	_	_	_	110	5.6	100.0	110	2.0	
NVT	_	_	_	162	8.2	100.0	162	3.0	
NTUI	1453	41.9	53.8	1250	63.1	46.2	2703	49.6	
NTUII	2018	58.1	81.4	460	23.2	18.6	2478	45.4	

** noNVT: Hospital without Neurovascular Treatment Team;*
NVT: Hospital with a Neurovascular Treatment Team;
NTUI: Neurovascular Treatment Unit level I;
NTUII: Neurovascular Treatment Unit level II.

Table S18. Characteristics of patients included in the stroke cohort in 2021 (PRE) from participating facilities according to in hospital readmissions within 30-days of discharge for ischemic stroke (Yes/No).

	**% of in hospital readmissions within 30-days of discharge for ischemic stroke**								**χ2 p-value**
	**Yes**			**No**			**Total**		
	**N**	**col%**	**row%**	**N**	**col%**	**row%**	**N**	**col%**	
**Total**	**182**			**2503**			**2685**		
**A&F intervention**									0.819
Exposed	93	51.1	6.9	1257	50.2	93.1	1350	50.3	
Control	89	48.9	6.7	1246	49.8	93.3	1335	49.7	
**Sex**								0.0	0.297
Female	76	41.8	6.2	1145	45.8	93.8	1221	45.5	
Male	106	58.2	7.2	1358	54.3	92.8	1464	54.5	
**Age (years)**								0.0	0.295
35-66	35	19.2	5.4	617	24.7	94.6	652	24.3	
67-76	57	31.3	8.0	657	26.3	92.0	714	26.6	
77-83	48	26.4	6.9	648	25.9	93.1	696	25.9	
84-100	42	23.1	6.7	581	23.2	93.3	623	23.2	
**Education level**								0.0	0.644
Bachelor's degree	19	10.4	7.0	254	10.2	93.0	273	10.2	
Lower middle high school	49	26.9	7.1	642	25.7	92.9	691	25.7	
Middle high school	39	21.4	5.9	619	24.7	94.1	658	24.5	
None or elementary	70	38.5	7.4	878	35.1	92.6	948	35.3	
Not stated	5	2.8	4.3	110	4.4	95.7	115	4.3	
**Concomitant clinical conditions**								0.0	
Cancer	9	5.0	9.5	86	3.4	90.5	95	3.5	0.287
Diabetes	6	3.3	5.1	112	4.5	94.9	118	4.4	0.454
Lipid metabolism disorders	4	2.2	8.3	44	1.8	91.7	48	1.8	0.665
Obesity	2	1.1	13.3	13	0.5	86.7	15	0.6	0.311
Obesity at index admission	3	1.7	4.0	72	2.9	96.0	75	2.8	0.332
Anaemias	3	1.7	3.9	74	3.0	96.1	77	2.9	0.307
Anaemias at index admission	1	0.6	1.5	67	2.7	98.5	68	2.5	0.078
Coagulation defects	_	_	_	1	0.0	100.0	1	0.0	0.787
Coagulation defects at index admission	1	0.6	10.0	9	0.4	90.0	10	0.4	0.685
Other haematological diseases	_	_	_	5	0.2	100.0	5	0.2	0.546
Other haematological diseases at index admission	_	_	_	16	0.6	100.0	16	0.6	0.279
Arterial hypertension	14	7.7	5.6	235	9.4	94.4	249	9.3	0.446
Previous myocardial infarction	3	1.7	5.8	49	2.0	94.2	52	1.9	0.770
Other forms of ischemic heart disease	9	5.0	7.8	106	4.2	92.2	115	4.3	0.648
Heart failure	12	6.6	9.8	111	4.4	90.2	123	4.6	0.179
Not well-defined forms and complications of heart disease	1	0.6	5.9	16	0.6	94.1	17	0.6	0.883
Rheumatic heart disease	1	0.6	7.1	13	0.5	92.9	14	0.5	0.957
Rheumatic heart disease at index admission	9	5.0	32.1	19	0.8	67.9	28	1.0	<.0001
Cardiomyopathies	_	_	_	14	0.6	100.0	14	0.5	0.312
Cardiomyopathies at index admission	_	_	_	12	0.5	100.0	12	0.4	0.349
Acute endocarditis and myocarditis	_	_	_	3	0.1	100.0	3	0.1	0.640
Other cardiac conditions	4	2.2	11.8	30	1.2	88.2	34	1.3	0.244
Other cardiac conditions at index admission	8	4.4	6.8	110	4.4	93.2	118	4.4	1.000
Conduction disorders and arrhythmias	13	7.1	8.2	145	5.8	91.8	158	5.9	0.455
Cerebrovascular diseases	20	11.0	10.6	169	6.8	89.4	189	7.0	0.031
Vascular diseases	3	1.7	5.6	51	2.0	94.4	54	2.0	0.718
Vascular diseases at index admission	4	2.2	3.6	106	4.2	96.4	110	4.1	0.181
Chronic obstructive pulmonary disease (COPD)	5	2.8	9.4	48	1.9	90.6	53	2.0	0.437
Chronic nephropathy	9	5.0	15.5	49	2.0	84.5	58	2.2	0.007
Chronic nephropathy at index admission	9	5.0	10.2	79	3.2	89.8	88	3.3	0.191
Chronic kidney disease	3	1.7	23.1	10	0.4	76.9	13	0.5	0.019
Diseases chronic diseases (liver, pancreas, intestine)	1	0.6	5.9	16	0.6	94.1	17	0.6	0.883
Diseases chronic diseases (liver, pancreas, intestine) at index admission	6	3.3	8.3	66	2.6	91.7	72	2.7	0.595
Cerebrovascular revascularization	_	_	_	15	0.6	100.0	15	0.6	0.295
Other heart surgery	4	2.2	14.8	23	0.9	85.2	27	1.0	0.095
Other vessel surgery	3	1.7	5.5	52	2.1	94.5	55	2.0	0.693
**Type of hospital***								0.0	0.591
noNVT	2	1.1	4.3	44	1.8	95.7	46	1.7	
NVT	8	4.4	10.1	71	2.8	89.9	79	2.9	
NTUI	87	47.8	6.6	1232	49.2	93.4	1319	49.1	
NTUII	85	46.7	6.8	1156	46.2	93.2	1241	46.2	

** noNVT: Hospital without Neurovascular Treatment Team;*
NVT: Hospital with a Neurovascular Treatment Team;
NTUI: Neurovascular Treatment Unit level I;
NTUII: Neurovascular Treatment Unit level II.

Table S19. Characteristics of patients included in the stroke cohort in 2022 (POST) from participating facilities according to in hospital readmissions within 30-days of discharge for ischemic stroke (Yes/No).

	% of in hospital readmissions within 30-days of discharge for ischemic stroke								χ2 p-value
	Yes			No			Total		
	N	col%	row%	N	col%	row%	N	col%	
**Total**	**210**			**2558**			**2768**		
**A&F intervention**									0.935
Exposed	108	51.4	7.5	1323	51.7	92.5	1431	51.7	
Control	102	48.6	7.6	1235	48.3	92.4	1337	48.3	
**Sex**									0.071
Female	83	39.5	6.6	1176	46.0	93.4	1259	45.5	
Male	127	60.5	8.4	1382	54.0	91.6	1509	54.5	
**Age (years)**									0.002
35-66	36	17.1	5.0	679	26.5	95.0	715	25.8	
67-76	44	21.0	7.0	583	22.8	93.0	627	22.7	
77-83	77	36.7	10.2	675	26.4	89.8	752	27.2	
84-100	53	25.2	7.9	621	24.3	92.1	674	24.3	
**Education level**									0.312
Bachelor’s degree	22	10.5	8.3	244	9.5	91.7	266	9.6	
Lower middle high school	47	22.4	6.0	742	29.0	94.0	789	28.5	
Middle high school	51	24.3	8.0	583	22.8	92.0	634	22.9	
None or elementary	79	37.6	8.6	841	32.9	91.4	920	33.2	
Not stated	11	5.2	6.9	148	5.8	93.1	159	5.7	
**Concomitant clinical conditions**									
Cancer	7	3.3	7.3	89	3.5	92.7	96	3.5	0.912
Diabetes	12	5.7	9.1	120	4.7	90.9	132	4.8	0.504
Lipid metabolism disorders	3	1.4	5.8	49	1.9	94.2	52	1.9	0.617
Obesity	2	1.0	11.8	15	0.6	88.2	17	0.6	0.514
Obesity at index admission	1	0.5	1.9	53	2.1	98.1	54	2.0	0.108
Anaemias	9	4.3	13.2	59	2.3	86.8	68	2.5	0.075
Anaemias at index admission	11	5.2	11.8	82	3.2	88.2	93	3.4	0.116
Coagulation defects	1	0.5	50.0	1	0.0	50.0	2	0.1	0.023
Coagulation defects at index admission	_	_	_	_	_	_	_	_	_
Other haematological diseases	1	0.5	11.1	8	0.3	88.9	9	0.3	0.689
Other haematological diseases at index admission	2	1.0	9.1	20	0.8	90.9	22	0.8	0.789
Arterial hypertension	21	10.0	7.9	246	9.6	92.1	267	9.6	0.857
Previous myocardial infarction	4	1.9	6.8	55	2.2	93.2	59	2.1	0.813
Other forms of ischemic heart disease	8	3.8	7.5	99	3.9	92.5	107	3.9	0.965
Heart failure	15	7.1	13.5	96	3.8	86.5	111	4.0	0.016
Not well-defined forms and complications of heart disease	3	1.4	15.0	17	0.7	85.0	20	0.7	0.209
Rheumatic heart disease	4	1.9	18.2	18	0.7	81.8	22	0.8	0.060
Rheumatic heart disease at index admission	1	0.5	3.4	28	1.1	96.6	29	1.0	0.398
Cardiomyopathies	1	0.5	7.7	12	0.5	92.3	13	0.5	0.989
Cardiomyopathies at index admission	3	1.4	14.3	18	0.7	85.7	21	0.8	0.245
Acute endocarditis and myocarditis	_	_	_	_	_	_	_	_	_
Other cardiac conditions	4	1.9	11.8	30	1.2	88.2	34	1.2	0.355
Other cardiac conditions at index admission	8	3.8	7.9	93	3.6	92.1	101	3.6	0.897
Conduction disorders and arrhythmias	17	8.1	10.8	141	5.5	89.2	158	5.7	0.121
Cerebrovascular diseases	12	5.7	6.6	171	6.7	93.4	183	6.6	0.586
Vascular diseases	8	3.8	11.3	63	2.5	88.7	71	2.6	0.235
Vascular diseases at index admission	7	3.3	5.7	115	4.5	94.3	122	4.4	0.430
Chronic obstructive pulmonary disease (COPD)	5	2.4	10.0	45	1.8	90.0	50	1.8	0.516
Chronic nephropathy	9	4.3	12.3	64	2.5	87.7	73	2.6	0.121
Chronic nephropathy at index admission	14	6.7	13.9	87	3.4	86.1	101	3.6	0.015
Chronic kidney disease	3	1.4	15.8	16	0.6	84.2	19	0.7	0.175
Diseases chronic diseases (liver, pancreas, intestine)	2	1.0	9.1	20	0.8	90.9	22	0.8	0.789
Diseases chronic diseases (liver, pancreas, intestine) at index admission	3	1.4	5.0	57	2.2	95.0	60	2.2	0.444
Cerebrovascular revascularization	_	_	_	12	0.5	100.0	12	0.4	0.320
Other heart surgery	3	1.4	11.5	23	0.9	88.5	26	0.9	0.445
Other vessel surgery	8	3.8	13.1	53	2.1	86.9	61	2.2	0.099
**Type of hospital***									0.624
noNVT	6	2.9	9.4	58	2.3	90.6	64	2.3	
NVT	8	3.8	9.6	75	2.9	90.4	83	3.0	
NTUI	110	52.4	7.9	1274	49.8	92.1	1384	50.0	
NTUII	86	41.0	7.0	1151	45.0	93.0	1237	44.7	

** noNVT: Hospital without Neurovascular Treatment Team;*
NVT: Hospital with a Neurovascular Treatment Team;
NTUI: Neurovascular Treatment Unit level I;
NTUII: Neurovascular Treatment Unit level II.
